# Effects of Yttrium Addition on the Microstructure Evolution and Electrochemical Corrosion of SN-9Zn Lead-Free Solders Alloy

**DOI:** 10.3390/ma14102549

**Published:** 2021-05-14

**Authors:** Wenchao Yang, Jun Mao, Yueyuan Ma, Shuyuan Yu, Hongping He, Da Qi, Yongzhong Zhan

**Affiliations:** 1 College of Materials Science and Engineering, Guangxi University, Nanning 530004, China; ywch053@163.com (W.Y.); 1815301030@st.gxu.edu.cn (J.M.); fishcat7@hotmail.com (Y.M.); qdksyjs2020@163.com (D.Q.); 2 Guangxi Key Laboratory of Processing for Non-Ferrous Metals and Featured Materials, MOE Key Laboratory of New Processing Technology for Non-Ferrous Metals and Materials, Nanning 530004, China; 3 Shenzhen Customs Industrial Products Inspection Technology Center, Shenzhen 518067, China; 4 College of Chemistry and Environmental Engineering, Shenzhen University, Shenzhen 518060, China; hehp@szu.edu.cn

**Keywords:** lead-free solder, Sn-Zn, electrochemical corrosion, microstructure evolution

## Abstract

Electrochemical corrosion behavior of ternary tin-zinc-yttrium (Sn-9Zn-xY) solder alloys were investigated in aerated 3.5 wt.% NaCl solution using potentiodynamic polarization techniques, and the microstructure evolution was obtained by scanning electron microscope (SEM). Eight different compositions of Sn-9Zn-xY (x = 0, 0.02, 0.04, 0.06, 0.08, 0.10, 0.20, and 0.30 wt.%) were compared by melting. The experimental results show that when the content of Y reached 0.06 wt.%, the grain size of Zn-rich phase became the smallest and the effect of grain refinement was the best, but there was no significant effect on the melting point. With the increases of Y content, the spreading ratio first increased and then decreased. When the content of Y was 0.06 wt.%, the Sn-9Zn-0.06Y solder alloy had the best wettability on the Cu substrate, which was increased by approximately 20% compared with Sn-9Zn. Besides, the electrochemical corrosion experimental shows that the Y can improve the corrosion resistance of Sn-9Zn system in 3.5 wt.% NaCl solution, and the corrosion resistance of the alloy is better when the amount of Y added is larger within 0.02–0.30 wt.%. Overall considering all performances, the optimal performance can be obtained when the addition amount of Y is 0.06.

## 1. Introduction

Sn-Pb solders have been widely used as interconnecting materials in electronic products because of their good physical condition and low cost [1,2]. However, lead and its compounds have high toxicity, which is harmful to the environment and human health. Lead-free solder must be researched to replace the traditional Sn-Pb solder. The most widely researched lead-free solder systems include Sn-Ag [3,4,5], Sn-Cu [6,7,8], Sn-Bi [9,10,11], and Sn-Zn [12,13,14,15] binary alloys and Sn-Ag-Cu [16,17], Sn-Zn-Al [18], Sn-Zn-Bi [19], and Sn-Zn-Ag [20] ternary alloys. The Sn-9Zn (198 °C) eutectic solder has become one of the most advantageous candidates, not only because of its excellent mechanical properties and close melting point to Sn-37Pb (183 °C), but also its rich reserves of Zn elements, low production cost, and nontoxicity. However, there are still practical challenges that need to be solved in application.

As one of the most promising representatives of lead-free solder, Sn-Zn has many unparalleled performance advantages and properties. However, due to the active chemical nature of zinc, it easily reacts with moisture and gas in air, resulting in corrosion problems. Sn-Zn is sensitive to chemical reactions with water and oxygen in the air, leading to corrosion and affecting practical applications. In the past few years, several researchers have studied Sn-Zn [21,22,23] systems, focusing on mechanical properties and wetting performance. However, there are relatively few studies on its corrosion performance. Thus, the corrosion behavior of Sn-Zn needs to be studied further. The alloying method is an effective way to obtain the refined microstructure of solders. For example, after adding Cr, Ti, and Al to the Sn-Zn alloy matrix, a refined microstructure can be obtained, and the oxidation resistance can be improved [12,24,25,26]. The addition of Ag to Sn-Zn can form uniformly dispersed Ag_5_Zn_8_ and AgZn_3_ phase matrices, which can effectively display a coarse, Zn-rich phase and improve the corrosion resistance of the solder [20]. Besides, introducing the Bi element into the Sn-Zn system can lower the melting point of the composite solder. However, an overdose doping of Bi will lead to the formation of a thick dendritic structure of the solder alloy and reduce the mechanical properties of the solder [19]. The rare earth (RE) elements have been considered as vitamins of metals, because a tiny amount of RE elements can significantly improve the properties of alloys. Zhang et al. [27] reported that the proper amount of Y can not only refine the structure of the Sn-Zn alloy but also improve the wettability and mechanical properties of the Sn-Zn/Cu solder joint and enhance the oxidation resistance of the solder alloy. Besides, as connecting material, the corrosion resistance of the solder plays a decisive role in the service life and safety of electronic products. Thus, in this study, the influence of Y on the electrochemical corrosion performance and microstructure evolution of Sn-9Zn-xY were investigated. 

## 2. Experimental Procedures

### 2.1. Material Fabrication

Sn-9Zn-xY composite solders were prepared by melting pure metals with a purity higher than 99.99%. The chemical composition table of solder alloy is shown in Table 1. First, the intermediate alloy Sn-1.5Y was prepared in a vacuum arc furnace (WK-Ⅱ, Physcience Opto-electronics Co., Ltd., Beijing, China) under an argon (Ar) atmosphere. Then, the intermediate alloy Sn-1.5Y, tin (Sn), and zinc (Zn) were weighed in accordance with the ratio and melted in a programmable heating furnace (SX2-10-12). The alloy was insulated at 350 °C for 1 h, and the surface was mantled with KCl-LiCl molten salt to prevent evaporation and oxidation. The melted alloy was poured into the mold and cooled naturally in the air to produce a composite solder alloy. The experiment mainly used X-ray Powder diffractometer (XRD), scanning electron microscope (SEM), Differential Scanning Calorimeter (DSC), the potentiodynamic polarization method, and cathode and anode extrapolation to study the properties of the alloy.

### 2.2. Microstructural Observation

The prepared sample was subjected to phase analysis. Using a Rigaku D/Max2500V X-ray diffractometer (Rigaku Corporation, Tokyo, Japan), the working voltage was 40 kV, the current was 100 mA, and the scanning angle was 20–80° at a rate of 8°/min.

The sample was embedded in epoxy resin, roughed by sandpaper 600 #–5000 #, and polished with 0.5 μm diamond paste. Then, the morphology of the sample was observed by SEM (Hitachi S-3400N, HITACHI, Tokyo, Japan).

### 2.3. Wettability Measurements

In the wettability experiment, we chose to use the copper substrate, which is a square copper sheet with a size of 40 mm × 40 mm × 2 mm. Above all, we needed to preprocess the copper substrate. First, it was polished with 1000 # water sandpaper. Then, ultrasonic cleaning was performed in acetone to remove oil stains. The copper substrate was rinsed with deionized water and dried. After rinsing with deionized water, the substrate was soaked in 10% HCl solution for 10 s to remove the surface oxide, rinsed thoroughly with deionized water, and blow-dried. Then the solder ball was placed in the center of the copper substrate, and the substrate was placed on an alumina oxide boat. After dropping the flux, the alumina oxide boat was smoothly placed into the resistance furnace for 250 °C, where it remained for 120 s. After taking it out, it was left to cool to room temperature and rinsed with water. According to the shape of the solder joint formed after the solder spreading solidification, the spreading rate *S_R_* was calculated by Equation (1):

(1) $$S_{R}=\frac{D-H}{D}\times 100\%$$

where *S_R_* is the spreading ratio, *H* is the height of the solder after the wetting test, and *D* is the diameter of the solder approximately as a sphere.

### 2.4. Melting Point Test

The melting points of the solder alloys were determined by DSC with the Synchronous Thermal Analyzer (Setaram Labsys Evo, SETARAM, Lyon, France). Approximately 30 ± 10 mg of the composite solder was placed in an alumina crucible under the protection of Ar atmosphere. The temperature of the instrument was first heated from room temperature to 150 °C at a heating rate of 10 °C/min. Then, it was heated to 250 °C at a rate of 5 °C/min.

### 2.5. Electrochemical Measurements

The composite solder wire was cut into a block with a cross-sectional area of 10 mm × 10 mm and a thickness of about 3 mm. The square section was polished with 3000 # as the working face, and the other side was pasted with copper paste, welded with copper wire as the lead, and sealed with paraffin. The reference electrode used in 3.5 wt.% NaCl solution was calomel electrode, and the auxiliary electrode used was platinum electrode. The CHI660D electrochemical analyzer (Shanghai Chenhua Instrument Company, Shanghai, China) was used to test the Sn-9Zn-xY sample with a scanning range of −2000 mV~500 mV and a scanning rate of 2 mV/s.

## 3. Results and Discussion

### 3.1. Microstructures of Sn-9Zn-xY Solder Alloys

**Figure 1:**
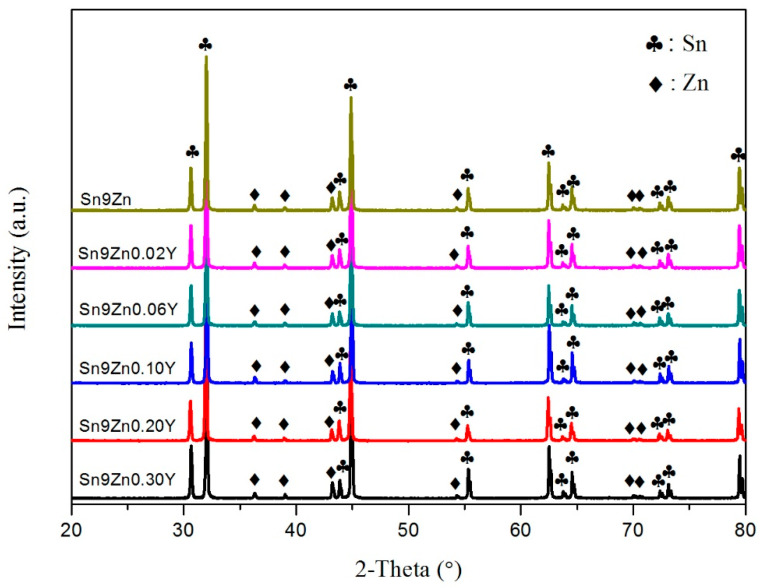
X-ray Powder diffractometer (XRD) diffraction pattern for Sn-9Zn-xY alloys (x = 0, 0.02, 0.06, 0.10, 0.20, 0.30 wt.%).

Figure 1 shows the XRD peaks of the Sn–9Zn-xY (x = 0, 0.02, 0.06, 0.10, 0.20, and 0.30 wt.%) alloys. Peaks were matched by Jade 6.0. Sn-9Zn-xY solder alloys were mainly composed of a large number of β-Sn phases and a small amount of Zn in a solid solution. However, after adding Y, the XRD spectra line of the solder alloy did not observe the other feature peaks. However, this does not mean that there was no new material formation, and material formation may be challenging to detect when the phase content in the alloy is very small. Therefore, it is possible that a new phase was produced, but that its content was small and could not be detected.

**Figure 2:**
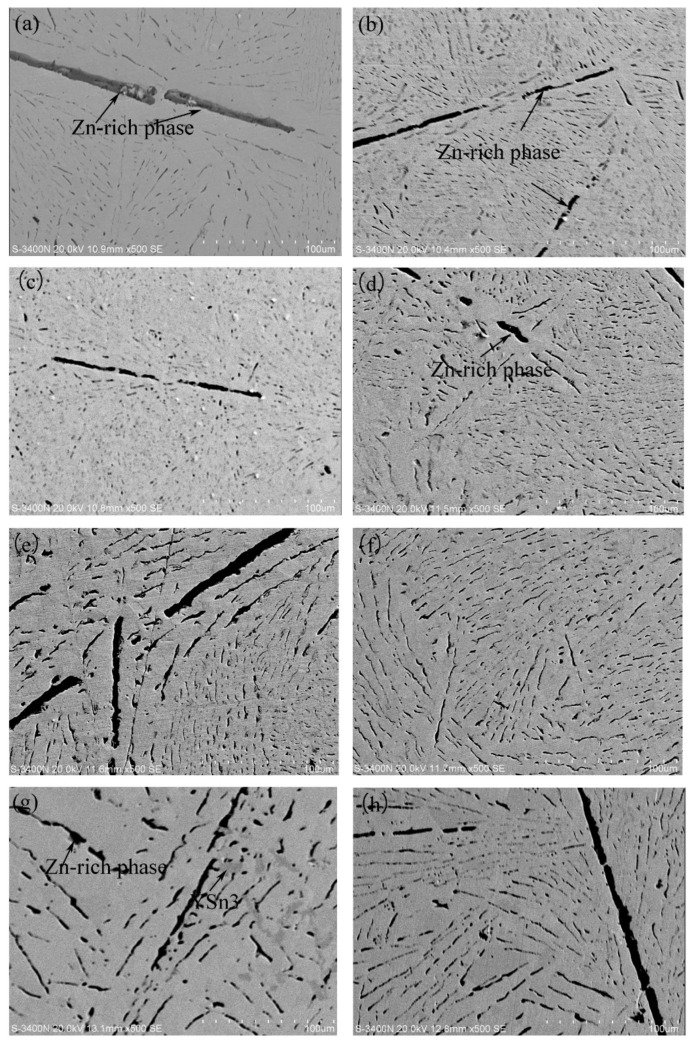
Scanning electron microscope (SEM) micrographs for (**a**) Sn-9Zn, (**b**) Sn-9Zn-0.02Y, (**c**) Sn-9Zn-0.04Y, (**d**) Sn-9Zn-0.06Y, (**e**) Sn-9Zn-0.08Y, (**f**) Sn-9Zn-0.10Y, (**g**) Sn-9Zn-0.20Y, (**h**) Sn-9Zn-0.30Y.

The SEM micrographs of the Sn-9Zn-xY alloys are shown in Figure 2. Figure 2a is the representative microstructure of eutectic Sn-Zn solder, which consists of two phases: The brighter large area is the matrix β-Sn phase, and the deeper coarse rod-like Zn phase is distributed on the Sn matrix. Adding Y to the Sn-9Zn alloy, as shown in Figure 2a–h, led to a significant change in the shape of the Zn-rich phase from the microscopic morphology. The strip shape was smaller than the original and was uniformly distributed in the matrix β-Sn. As the Y content increased, the rod-like Zn-rich phase was further refined. When the doping level was 0.06 wt.%, as shown in Figure 2d, the Zn-rich phase in the alloy system was most uniformly distributed, and the size became finer. Compared with the Zn-rich phase in Sn-9Zn as Figure 2a, the refinement was very significant. However, as the content of Y continued to increase and reached 0.2 wt.%, as shown in Figure 2g, a new phase with a darkness between the Sn matrix and the Zn-rich phase appeared in the alloy, and scattered distribution was observed on the Sn matrix. This compound is YSn_3_. The binary Sn-Y [28] phase diagram is used to show that YSn_3_ is unstable at a high temperature and decomposes at 515 °C. The melting point of the rare earth compound is generally high, and trace rare earth elements are present in the molten metal alloy and can be adsorbed on the surface of the grain boundary and prevent the growth of the crystal grains to achieve the effect of refining the tissue. At the same time, the rare earth compound can also reduce the surface activity point and improve the phase interface, uniformity of the surface, fluidity of the molten metal, and compactness of the microstructure.

### 3.2. Wettability Analysis

**Figure 3:**
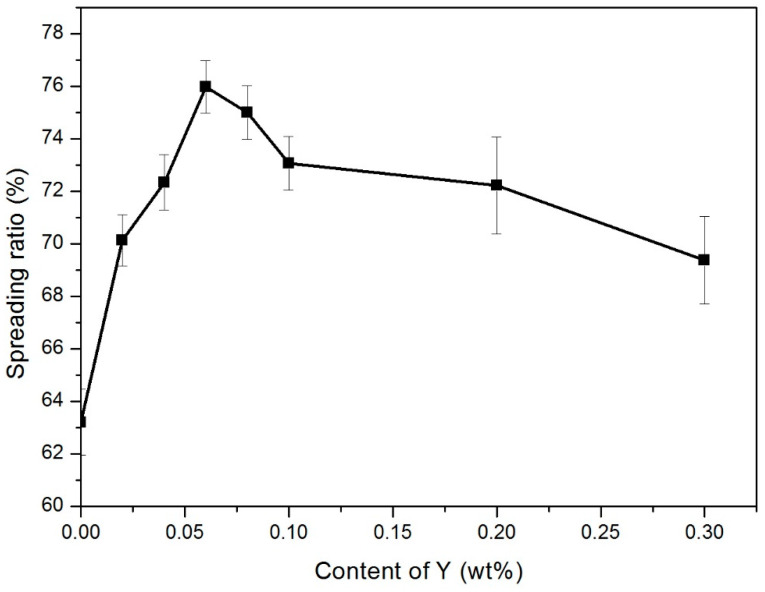
Spreading ratio curve of Sn-9Zn-xY alloys.

The spreading ratio is shown in Figure 3. As we can observe from curve, the addition of Y had a significant effect on the wettability of Sn-9Zn alloy system. When Y was added to the alloy, the spreading ratio of Sn-9Zn solder increased first and then decreased with the increase of Y content. When the Y content was 0.06 wt.%, the spreading rate reached 76%, and the best wettability was obtained. However, when the amount of Y added continued to increase, the spreading ratio dropped sharply, and the wetting performance deteriorated.

Zn is an active metal element in Sn-9Zn. At 250 °C, the oxidation of the Zn leads to the formation of oxides that are difficult to be wetted on the Cu substrate, resulting in poor wettability of the alloy solder. Y is a rare earth element, and its properties are relatively lively. Adding a trace amount can obviously affect the microstructure of the alloy. When a small amount of Y is added, the original Zn-rich phase in Sn-9Zn is refined, and Zn is evenly distributed in the matrix so that the Zn in the matrix no longer polymerizes in a simple form. At high temperatures, the degree of oxidation is reduced compared to when it is not refined, so the wetting performance is increased. The refinement is most obvious when the content of Y is 0.06 wt.%, so the oxidation degree of Zn is the smallest and the wetting performance is the best. However, when a larger amount of Y is added, the active compound YSn_3_ in the matrix will also generate oxides that are not easy to wet with the base metal copper plate at high temperatures. Thus, the spreading rate of the solder is reduced, and the wettability of the solder alloys and the base metal copper plate is deteriorated. In summary, with the increase of Y content, there is a certain refinement effect on the crystal grain, but the increase in wettability is mainly due to the surface activity of rare earth elements, which are easier to agglomerate at the solder/flux interface in the molten state, reducing the liquid solder. The interface surface tension between the material and the substrate accelerates the wetting of the brazing alloy to the copper substrate [29,30,31]. However, excess Y can also cause a decrease in solderability, which is caused by an increase in the viscosity of the solder. When the Y content is too high, it easily forms oxides, but it increases the surface tension and deteriorates the wetting behavior.

### 3.3. Melting Temperature

**Figure 4:**
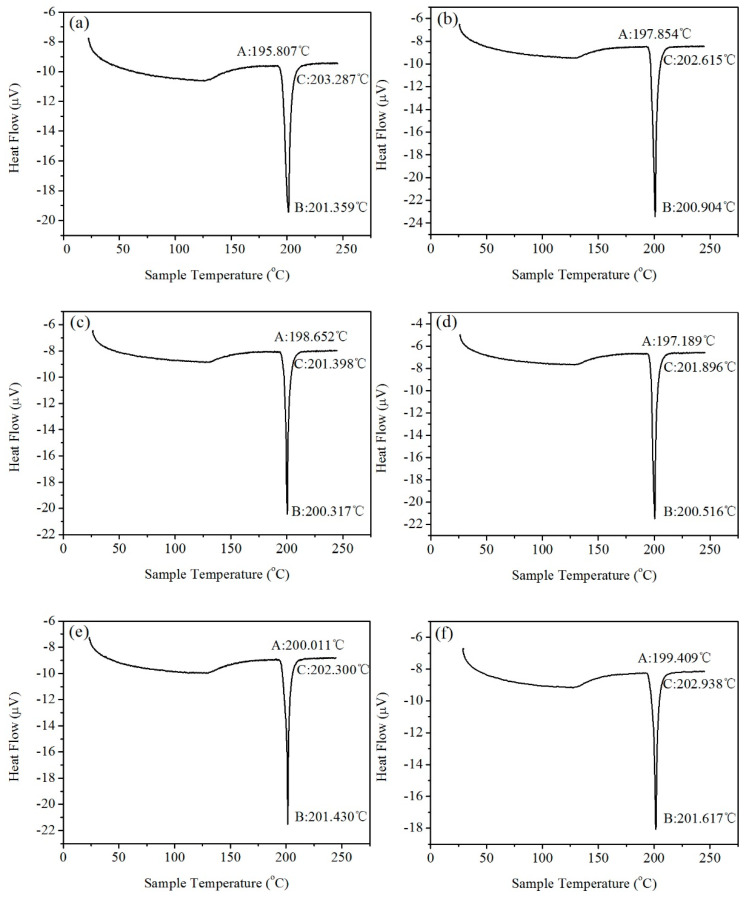

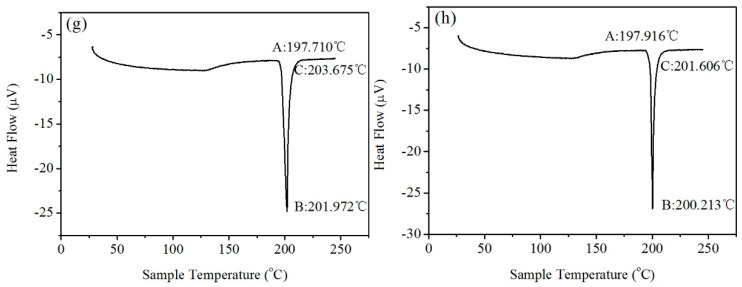
The Differential Scanning Calorimeter (DSC) curves of Sn-9Zn-xY alloys: (**a**) Sn-9Zn, (**b**) Sn-9Zn-0.02Y, (**c**) Sn-9Zn-0.04Y, (**d**) Sn-9Zn-0.06Y, (**e**) Sn-9Zn-0.08Y, (**f**) Sn-9Zn-0.10Y, (**g**) Sn-9Zn-0.20Y, (**h**) Sn-9Zn-0.30Y.

Figure 4 shows the DSC curve of Sn-9Zn-xY solder alloys. From the overall curve, during the heating process of the metal, the alloy had an endothermic reaction near the melting point. Because the metal underwent a phase transition reaction during the melting process to absorb heat, and there was a peak of depression. As can be seen from Figure 4, the sample underwent endothermic reaction at point A, and its heat flux dropped sharply. Point A defines the starting point of alloy melting, which is the solidus temperature. As the sample temperature continued to increase, the sample underwent a eutectic melting reaction within a certain range after point A, and the reaction endothermic heat flow showed a downward peak. Point B is the peak of the endothermic peak, and the temperature corresponding to this peak is the melting point. The melting process of most alloys occurs in the temperature range from point A to point C. When the temperature rises beyond point C, the heat flow flattens out, the metal is completely liquefied, and the melting process ends.

**Figure 5:**
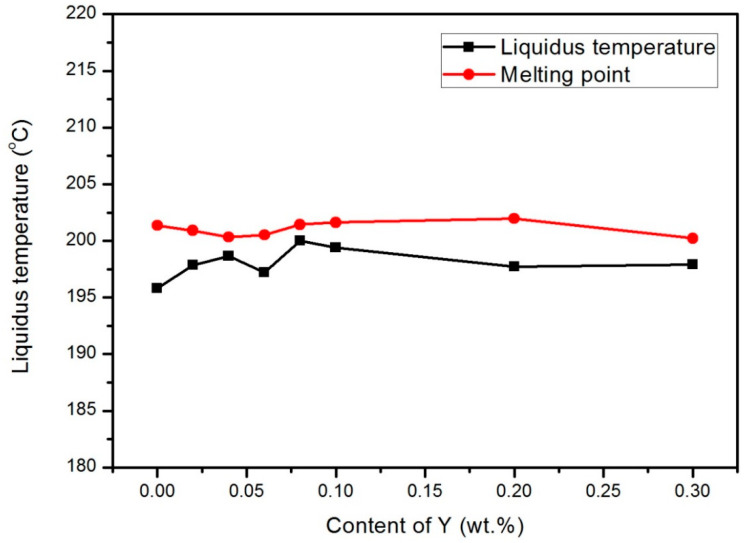
Melting point and melting range curves of the Sn-9Zn-xY solder alloys.

The initial melting temperature, melting point, and melting range of the Sn-9Zn-xY solder alloys are listed in Table 2. The melting point and melting range curve of the Sn-9Zn-xY solder alloys are shown in Figure 5. According to the analysis in Table 2 and Figure 5, the melting point of Sn-9Zn-0.30Y was the lowest, but only about 1 °C lower than that of Sn-9Zn. Moreover, the melting process of Sn-9Zn-0.08Y was the lowest, but only about 3 °C lower than that of Sn-9Zn. These small changes indicate that the amount of Y added does not have a great influence on the melting point and melting range of the alloy. A small amount of Y, Sn, and Zn do not generate high melting point compounds. In turn, the melting point of the alloy does not increase, nor is the melting point significantly reduced. When adding Y content to Sn-9Zn in the range of 0–0.3 wt.% to improve other properties, Y will not have significant influence on the melting properties of the Sn-9Zn solder alloys.

### 3.4. Electrochemical Behavior

**Figure 6:**
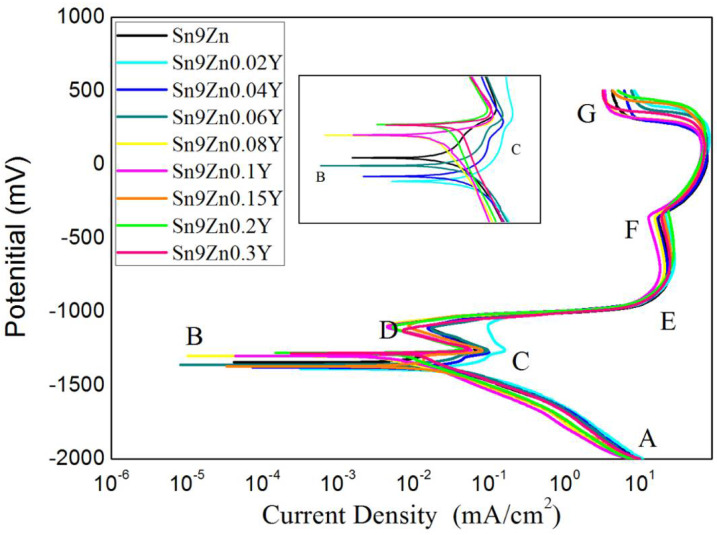
Potentiodynamic polarization curves of Sn-9Zn-xY solders in 3.5 wt.% NaCl solution.

The Sn-9Zn-xY solder alloys was studied by potentiodynamic polarization to determine the effect of varying Y content on the corrosion resistance. Figure 6 shows the electric polarization plots of Sn-9Zn-xY (x = 0, 0.02, 0.04, 0.06, 0.08, 0.10, 0.20, 0.30 wt.%) solders in 3.5 wt.% NaCl solution. The polarization curve in the range of −2000 mV to +500 mV at a scanning speed of 2 mV/s was investigated.

In the cathode region AB, the reaction taking place is the reduction of water, that is, the formation of hydrogen (H_2_) and hydroxyl ions (OH^−^). During this experiment, hydrogen bubbles were observed on the surface of the sample. The reaction is as follows:

2H_2_O + 2e^−^→H_2_ + 2OH^−^ (2)

At point B, because the activity and standard electrode potential of Zn were lower than Sn and Y, Zn preference occurred the dissolution reaction [32,33].

Zn + 2OH^−^→Zn(OH)_2_ + 2e^−^ (3)

Zn + 2OH^−^→ZnO+H_2_O + 2e^−^ (4)

The values of the potentials for reactions (3) and (4) approached the E_corr_ values listed in Table 3.

As the potential continued to increase, the active dissolution of zinc continued to point C, where the concentration reached a critical value and an ultrafine microstructure was produced on the solder surface. It was found that the current density decreased as the electric potential increased during region CD, which was created by the active dissolution of Sn according to the reactions below:

Sn + 2OH^−^→Sn(OH)_2_ + 2e^−^ (5)

Sn(OH)_2_→SnO + H_2_O (6)

Abayarathna et al. [34] reported that the formation of zincate membranes on regional CD has no protective effect, and that the formation of the membranes is a process limiting transport.

The currents sharp increases during region DE may indicate more serious corrosion and weaken of the ability to form a passive film. Passivation for the solder alloys began at point E. From point E to point F, the current density was nearly independent of the potential. It is generally believed that zinc passivation begins with the precipitation of zincate ions in the form of Zn (OH)_2_ or ZnO. The deposition of ZnO or Zn (OH)_2_ on the surface hinders the dissolution of the active sites and passivates part of the surface.

A sharp increase in current density was observed at about -300mV (point F). Due to the incorporation of Cl^−^ ions into the oxide layer, the film began to break at point F. The potential at F point is called the breakdown potential, which is also known as the pitting potential. Under the action of pitting, certain points on the passivated film become part of the destruction. Therefore, the anode current density increased sharply.

**Figure 7:**
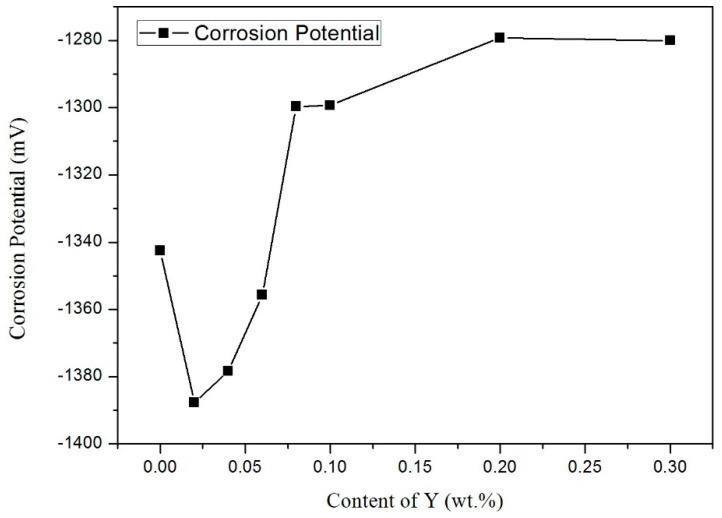
Effect of Y content on the E_corr_ value obtained during potentiodynamic polarization of Sn-9Zn-xY in 3.5 wt.% NaCl solution.

Table 3 shows the electrochemical parameters, including the corrosion potential (E_corr_), corrosion current density (I_corr_), and corrosion rate. These parameters were obtained by fitting the polarization curve with Cview software. Figure 7 shows the change trend of the influence of different contents of Y on the self-corrosion potential. As shown in Figure 7 and Table 2, the E_corr_ values decreased when the addition of Y content only was 0.02 wt.%. However, with the increase of Y content, Ecorr showed a tendency toward more negative potentials. The E_corr_ of Sn-9Zn-0.06Y was close to the E_corr_ of Sn-9Zn. With a continued increase in Y content, the E_corr_ value increased, reaching a value higher than the E_corr_ of Sn-9Zn. Then, value remained stable with the increase of increasing Y content until the Y reached a certain value. However, I_corr_ value decreased after initially increasing and then leveled off. The greatest influence on the corrosion resistance was the active Zn in Sn-9Zn-xY alloys. Zn was active and easily oxidized when it existed as Zn-rich phase. Y refined the rich Zn phase, homogenized it, and reduced the probability of Zn corrosion on the surface of the sample to improve the corrosion resistance of the alloy. At the same time, Y did not generate the active compound in the alloys. A recent report by Ralston et al. [35] showed that, when testing systems with a certain degree of passivation, the corrosion rate decreases as the grain size decreases. Osório et al. [36] also proved that the corrosion behavior of Sn-Ag solder alloy is closely related to the structure of the array. Therefore, the addition of trace Ti increased the corrosion resistance of Sn-9Zn alloy due to the structural deterioration of the alloy, that is, a large number of primary zinc-rich precipitates were eliminated, and the eutectic structure become more uniform. Liu et al. [26] reported that the addition of Ti led to zinc-rich precipitation phase refinement in the structure, which is conducive to the formation of a more protective passivation film on the surface of the modified composite solder and the enhancement of the corrosion resistance of the material.

## 4. Conclusions

1. The addition of Y refined the Zn-rich phase of the matrix and made the Zn phase narrow and uniformly distributed. When the addition of Y was 0.06 wt.%, it showed the most significant refinement effect. However, when the content of Y continued to increase, YSn_3_ was unstable at high temperature in the matrix.
2. The spreading rate of Sn-9Zn solder increased with the addition of Y. When the Y content was 0.06 wt.%, the wettability was the best. However, when the amount of Y added continued to increase, the wetting performance dropped sharply.
3. The amount of Y added had no significant influence on the melting point and melting range of the alloy.
4. Adding Y to Sn-9Zn improved the corrosion resistance of solder. When a small amount of Y was added, the corrosion resistance of the alloy was lower than that of Sn-9Zn. However, with the increase of Y content, the corrosion potential increased. When Y content was 0.06 wt.%, the corrosion potential of the alloy was close to that of the Sn-9Zn alloy. The corrosion potential increased with the increase of Y content, which was higher than that of Sn-9Zn, and tended to a stable value. The addition of Y can refine the Zn-rich phase of Sn-9Zn system and improve the corrosion resistance of the alloy.

## Figures and Tables

**Table 1:** Chemical compositions of the Sn-9Zn-XY alloy (wt.%).

Solder Alloy	Sn (wt.%)	Zn (wt.%)	Y (wt.%)
Sn9Zn	91.00	9	0
Sn9Zn0.02Y	90.98	9	0.02
Sn9Zn0.04Y	90.96	9	0.04
Sn9Zn0.06Y	90.94	9	0.06
Sn9Zn0.08Y	90.92	9	0.08
Sn9Zn0.10Y	90.90	9	0.1
Sn9Zn0.20Y	90.80	9	0.2
Sn9Zn0.30Y	90.70	9	0.3

**Table 2:** DSC results of the Sn-9Zn-xY solder alloys.

Solder Alloy	Solid Phase Line, °C	Melting Point, °C	Melting Range, °C
Sn9Zn	195.8	201.4	5.6
Sn9Zn0.02Y	197.8	200.9	3.1
Sn9Zn0.04Y	198.7	200.3	1.6
Sn9Zn0.06Y	197.2	200.5	3.3
Sn9Zn0.08Y	200.0	201.4	1.4
Sn9Zn0.10Y	199.4	201.6	2.2
Sn9Zn0.20Y	197.7	201.9	4.2
Sn9Zn0.30Y	197.9	200.2	2.3

**Table 3:** The electrochemical corrosion parameters of Sn-9Zn-xY solder alloys in 3.5 wt.% NaCl solution.

Y (wt.%)	I_corr_ (mA/cm^2^) × 10^−3^	E_corr_ (mV)	Corrosion Rate (mm/a)
0	3.81	−1342.6	0.096
0.02	39.6	−1387.7	0.996
0.04	21.6	−1378.3	0.548
0.06	7.43	−1355.7	0.188
0.08	2.09	−1299.7	0.053
0.1	3.27	−1299.3	0.083
0.2	4.39	−1279.3	0.111
0.3	5.27	−1280.1	0.133

## Data Availability

Data is contained within the article.
